# Temporal patterns in *count-to-ten* fetal movement charts and their associations with pregnancy characteristics: a prospective cohort study

**DOI:** 10.1186/1471-2393-12-124

**Published:** 2012-11-06

**Authors:** Brita Askeland Winje, Jo Røislien, J Frederik Frøen

**Affiliations:** 1 Division of Epidemiology, Norwegian Institute of Public Health, PO Box 4404 , Nydalen, 0403, Oslo, Norway; 2 Department of Biostatistics, Institute of Basic Medical Sciences, University of Oslo, Oslo, Norway

**Keywords:** Fetal movement, Kick counting, Decreased fetal movement, Functional data analysis, Principal components, Temporal pattern

## Abstract

**Background:**

Fetal movement counting has long been suggested as a screening tool to identify impaired placental function. However, quantitative limits for decreased fetal movement perform poorly for screening purposes, indicating the need for methodological refinement. We aimed to identify the main individual temporal patterns in fetal movement counting charts, and explore their associations with pregnancy characteristics.

**Methods:**

In a population-based prospective cohort in Norway, 2009–2011, women with singleton pregnancies counted fetal movements daily from pregnancy week 24 until delivery using a modified "count-to-ten” procedure. To account for intra-woman correlation of observations, we used functional data analysis and corresponding functional principal component analysis to identify the main individual temporal patterns in fetal movement count data. The temporal patterns are described by continuous functional principal component (FPC) curves, with an individual score on each FPC for each woman. These scores were later used as outcome variables in multivariable linear regression analyses, with pregnancy characteristics as explanatory variables.

**Results:**

Fetal movement charts from 1086 pregnancies were included. Three FPC curves explained almost 99% of the variation in the temporal data, with the first FPC, representing the individual overall counting time, accounting for 91% alone. There were several statistically significant associations between the FPCs and various pregnancy characteristics. However, the effects were small and of limited clinical value.

**Conclusions:**

This statistical approach for analyzing fetal movement counting data successfully captured clinically meaningful individual temporal patterns and how these patterns vary between women. Maternal body mass index, gestational age and placental site explained little of the variation in the temporal fetal movement counting patterns. Thus, a perceived decrease in fetal movement should not be attributed to a woman’s basic pregnancy characteristics, but assessed as a potential marker of risk.

## Background

Fetal movement (FM) counting by pregnant women has long been suggested as a screening tool to identify impaired placental function. The rationale is that a fetus will respond to reduced uteroplacental blood flow and fetal hypoxia by decreasing gross fetal movements [1]. Decreased fetal movement (DFM) is associated with placental pathologies [2,3] and a range of adverse pregnancy outcomes, including fetal growth restriction and death [4-8]. If DFM is recognized early and managed appropriately, adverse outcomes may thus be prevented [9].

So far, however, there is no conclusive evidence to support or refute formal FM counting as a means to reduce perinatal morbidity and mortality [10-15]. Despite this, extensive self-screening for DFM continues and management of maternal concerns for DFM remain a challenge in obstetric care [16-18]. Indeed, in a recent Lancet series of stillbirth prevention increased awareness and timely evaluation of women reporting DFM was ranked among top research priorities by an expert panel [10]. Thus, ways to improve our understanding of relevant temporal patterns in FM counting data and what changes in FM counting that are clinically relevant, are urgently needed.

The traditional approach to analyzing FM charts has been to focus on point-wise, i.e. day-to-day, group averages based on birth outcome [19-24] and deviations from these averages compared to fixed quantitative DFM alarm limits. This is problematic for several reasons. A group average is not representative of individual behavior. Further, the strong correlation between observations from the same woman is not taken into account, and individual temporal patterns are consequently lost. Such temporal patterns, e.g. emerging trends, shifts and changes in variability, may hold valuable information on the nature of FM counting. By focusing on fixed alarm limits this individual, temporal information may be overlooked.

In order to uncover temporal patterns on an individual level, rather than merely look at day-to-day group averages, we turn to functional data analysis (FDA), a statistical methodology specifically developed for analyzing curve data, and long time series observations [25]. FDA properly adjusts for the intra-woman correlation between measurements. We apply FDA, and the corresponding functional version of principal component analysis, to analyze FM count data from a Norwegian prospective cohort study of women with singleton pregnancies. To our knowledge, this is the first application of FDA on FM count data.

In a prospective screening scenario, birth outcomes are not yet known. To eventually be able to single out pathological patterns in FM counting, an important initial step is to identify the main temporal patterns in FM count data from a total population, and establish whether the expected temporal pattern of a given woman’s FM count data depends on basic pregnancy characteristics such as maternal body mass index, gestational age and placental position. If so, what is the effect and what are the implications?

The aim of this study was to identify the main temporal patterns in FM count data on an individual level in pregnancies recruited from a total population. We also wanted to explore whether any of these temporal patterns were associated with basic pregnancy characteristics. To the best of our knowledge, our study is the first to extract individual temporal patterns from FM chart data.

## Methods

### Details of ethical approval

Written informed consent was obtained from all participants. The study was approved by the Regional Committee for Medical Research Ethics, S-08694d, 2008/18353, 06.26.2009.

### Data collection

From July 2009 to July 2011, all women with singleton pregnancies attending Østfold Hospital Trust for routine ultrasound screening in pregnancy week 17–19 were invited to participate in the study. This routine ultrasound screening captures > 98% of the pregnant population [26]. A designated research midwife provided the women with information about FM counting and how to use and interpret the FM chart described below. Demographic and obstetric information was obtained from antenatal pregnancy charts and hospital records. Women with pregnancies under consideration for termination at the time of recruitment and women who could not speak sufficient Norwegian to read and understand the study protocol were excluded from the study.

During the two-year period 2468 women (41% of eligible pregnancies) agreed to participate in the study and gave written informed consent. Among them 1445 (59%) later submitted their FM charts and constitute the study sample, see flowchart for recruitment (Additional file 1: Figure S1). We excluded 359 FM charts (25%) from analyses due to missing counting observations (details below), leaving 1086 women in the final sample. Demographic and obstetric characteristics for the total pregnant population at Østfold Hospital Trust (data from Medical Birth Registry of Norway, year 2009 used as reference [27]) and women included in the present analysis are presented in Table 1. Compared to the total population of pregnant women at Østfold Hospital Trust, our sample for the present analysis included more primiparous women, fewer smokers, fewer cesarean sections, and fewer preterm and low birth weight babies. A broadly similar bias existed for all subsamples when compared to the total population. 

**Table 1:** Demographic and obstetric characteristics

	**Study group, n= 1086**	**Østfold Hospital, year 2009**^**#**^, **n= 3212**	**Relative risk (RR)**	**p**^**//**^
	n (%)**	n (%)**	**RR (95% CI)**	
**MATERNAL CHARACTERISTICS**				
Maternal age, years [mean, SD]	30.7 [4.7]	29.2 [5.2]		
Maternal age ≥ 35 years	196 (18.0)	544 (16,9)	1.1 (0.9-1.2)	0.400
Maternal BMI, kg/m^2^, [mean, SD]	24.8 [5.1]	Not available	-	-
Maternal obesity (BMI ≥ 30 kg/m^2^)	155 (14.3)	Not available	-	-
Primiparity	574 (52.9)	1370 (42.7)	1.2 (1.2-1.3)	<0.001
Daily/occasionally smoking 1.trimester	93 (8.5)	673 (21.0)	0.4 (0.3-0.5)	<0.001
Anterior placental site	479 (44.1)	Not available	-	-
**DELIVERY MODE**				
**Vaginal deliveries**	905 (83.3)	2545 (78.3)	1.1 (1.0-1.1)	<0.001
Induced vaginal deliveries	182 (20.1)	304 (11.9)	1.7 (1.4-2.0)	<0.001
Assisted vaginal delivery	131 (14.5)	282 (11.1)	1.3 (1.1-1.6)	0.006
**Cesarean sections (CS), total**	181 (16.7)	706 (21.6)	0.8 (0.7-0.9)	<0.001
Emergency CS	117 (64.6)	442 (62.6)	1.0 (0.9-1.7)	0.552
**CHARACTERISTICS OF THE NEWBORNS AND BIRTH OUTCOME**				
Gestational age, weeks [mean,SD]	39.6 [1.6]	39.2 [1.9]		
Male gender	562 (51.7)	1726 (52.7)	1.0 (0.9-1.1)	0.588
Birth weight in grams [mean, SD]	3584 [524]	3492 [623]	-	-
Low birth weight (<2500gr)	27 (2.5)	147 (4.5)	0.6 (0.4-0.9)	0.006
Stillbirth [> 22 weeks, per 1000]	3 [2.7/1000]	8 [2.4/1000]		
Preterm (22^0^-36^6^ weeks)	52 (4.8)	213 (6.6)	0.7 (0.5-1.0)	0.031
Apgar score <7_5min_	16 (1.5)	55 (1.7)	0.9 (0.5-1.5)	0.642

^#^*Data from Medical Birth Registry of Norway (MBRN) (*http://mfr-nesstar.uib.no/mfr/*, Des 19. 2011), year 2009 was latest available complete update and is used as reference, denominator varies between subgroups – denominator reflects number of deliveries (n=3212) or number of infants (n=3275)*.

^**//**^*Chi-square test for categorical data*.

^**^Number and proportion unless other is specified in the left column.

The table presents demographic and obstetric characteristics and birth outcome of the total population of pregnant women in Østfold Hospital and for the women included in the analyses.

### Fetal movement counting and recording

The counting protocol (Additional file 2: Appendix 1) is a continuation of the protocol from the international collaboration Fetal Movement Intervention Assessment (FEMINA) [16-18]. Participating women were from pregnancy week 24 asked to count FM in a daily time to “count-to-ten” procedure, within a preferred two-hour time period chosen by the mother, when she knew her baby was usually active. She was instructed to focus on FM, preferably lying down, and to initiate counting when she perceived the first movement, indicating that her baby was awake, and record the time until she had counted the additional nine. All movements counted as kicks. Simultaneous, rolling movements counted as one. Hiccups were disregarded. The mother then recorded the counting time in the FM chart (Additional file 3: Appendix 2). The counting protocol is an adjusted version of the time to “count-to-ten" methodology using focused counting [14]. Further details and the rationale for the counting method have been presented previously [28]. The FM counts from the last 90 days before birth were included in the analysis.

### Missing data in the FM charts

Overall, the 1445 women who submitted their FM charts recorded counting in 77% of days from week 24 until birth, 80% and 59% in the preterm and term period respectively. In many of the FM charts a substantial amount of counting observations was missing. The last 90 days preceding birth only 120 (8%) women had complete FM charts; 518 (36%) had 1–10 percent missing, 448 (31%) had 11–50 percent missing and 359 (25%) had more than 50% missing.

The unit of observation in our analyses is the individual FM chart. Since more than 90% of FM charts had some missing, imputation was necessary before proceeding with further statistical analysis. We chose to include only women with on average more counting days per week than not, i.e. at least 4/7 = 57% of the last 90 days preceding birth. This left 1086 (75%) women for statistical analysis. Comparing demographic and birth outcomes between women who were excluded due to missing observations and those included in the analyses, showed no difference between the groups other than a lower proportion women aged ≥ 35 years among those excluded (Additional file 4: Table S1).

### Statistical analyses

Descriptive statistics are presented as mean, standard deviation (SD) and range, or frequency and percentage (%). The FM counting observations are heavily skewed, and were log transformed before further statistical analysis. Statistical analysis was performed in SPSS 12.0, R 2.12 and Winbugs 3.0. See Appendix A for details.

#### Outliers

DFM is often perceived as extreme changes in FM by the mother, and several of the FM charts included such extreme counts reflecting comparably long counting times relative to the body of the woman’s observations. The aim of the current study was to extract the general temporal patterns, and outlying observations were thus removed before further statistical analysis.

#### Functional data analysis

The FM charts were analyzed using functional data analysis (FDA), a statistical methodology specifically developed for analyzing curve data or long time series [25]. In applying FDA, a continuous, smooth curve is fitted to each woman’s FM count series, and statistical analysis is then performed on these fitted curves rather than on the actual FM counts. In this manner intra-woman correlation of observations is accounted for. The smoothing removes natural day-to-day variation, i.e. measurement error and normal fluctuations in fetal activity, leaving the overall individual temporal behavior for statistical analysis. The 95% credibility intervals (Crls) are the Bayesian parallel to confidence intervals (CI) to assess estimation uncertainty in the fitted curves [29].

#### Functional principal component analysis

Principal component analysis (PCA) is a statistical methodology that can be seen as unveiling the internal structure of the data in a way that best describes the variation in the data [30]. In order to identify common temporal patterns between the individually fitted smooth curves, we used functional principal component analysis (FPCA) [31]. FPCA stands in direct parallel to traditional PCA. The result of an FPCA is a set of functional principal component curves (FPC) describing the main temporal patterns. Each woman is provided with a score on each of the FPC curves, representing to what degree that specific pattern is present in her fitted smooth FM curve. Women with close-to-zero FPC scores have FM charts that are similar to the overall temporal mean. Similarly we also ran FPCA for the deviations between the woman's actual individual counting data and her individual fitted FDA curves (residuals) so as to explore the effect of individual day-to-day variation.

#### Multiple regression models

To explore the effects of normal variants of basic pregnancy characteristics on the temporal FM patterns, the FPC scores were used as outcome variables in univariate and multiple linear regression analyses. Body Mass Index (BMI) was included as a categorical variable according to WHO criteria [32]; reference group (BMI<25), overweight (25≤BMI<30), and obesity (30≤BMI) and anterior placental site (predominantly non-anterior/anterior) and parity (multiparity/primiparity) was included as dichotomous explanatory variables. Since we aligned our data from birth and 90 days backwards, we adjusted for pregnancy length by including gestational age as a continuous explanatory variable. Similar regression analyses were performed for the FPC for residuals. P-values below 0.05 were considered statistically significant.

## Results

**Figure 1:**
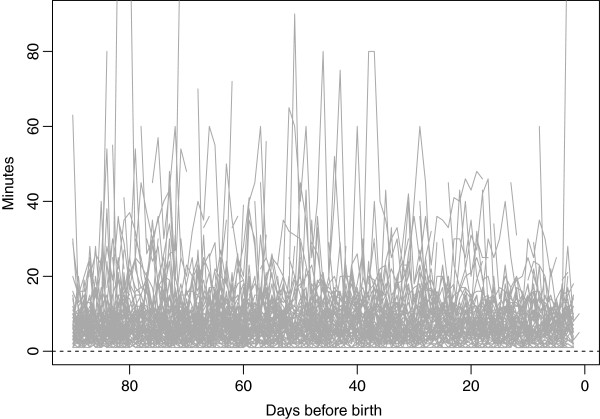
Original fetal movement count data from a random sample of 100 fetal movement charts.

**Figure 2:**
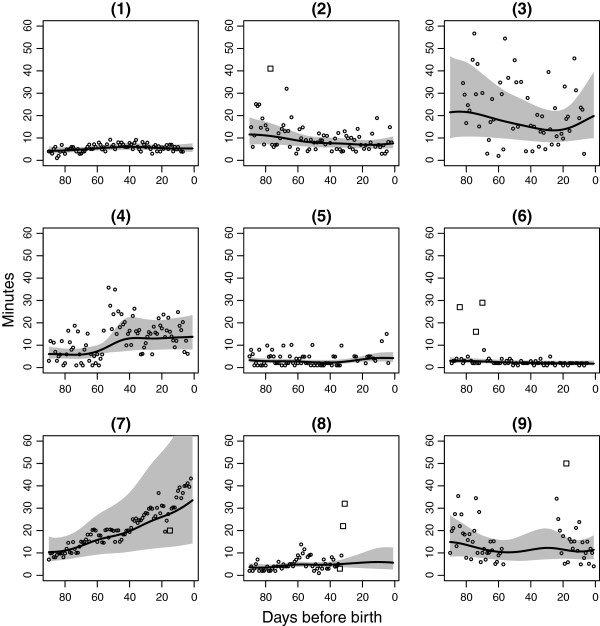
**Smooth curve fits for nine randomly selected fetal movement charts.** Transformation of observed fetal movement count data (dots) to smooth curve fits (solid line), together with removed outlying observations (squares) for 9 randomly selected women. Grey shaded area is 95% CrI for the fit.

FM count data for a random sample of 100 women is shown in Figure 1. Individually fitted, smooth curves with 95% CrI superimposed for nine women are shown in Figure 2. In general, the fitted curves, smoothing out natural day-to-day variation, were for most women largely horizontal, with little variation relative to their individual temporal mean. Note how the CrIs are wider in the presence of missing observations.

**Figure 3:**
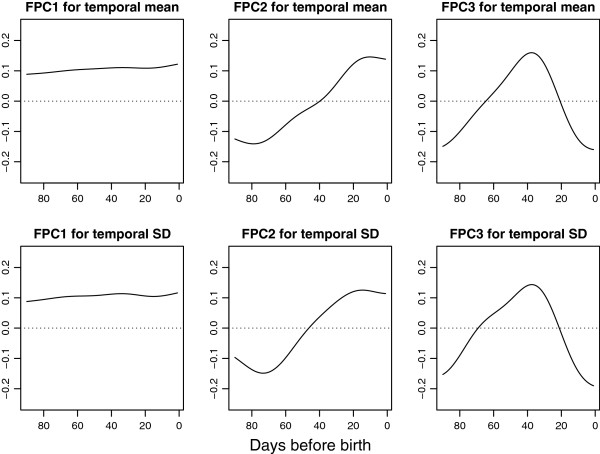
**The first three functional principal component curves for mean and residuals.** The first three functional principal components (FPC) for mean (upper row) and residuals (lower row). The effect of the FPC scores is on a multiplicative scale. The analysis of FM count data were on a log-transformed scale (additive effect) which later has been anti-logged (multiplicative effect). Zero score on a component therefore corresponds to multiplying by one and the mean score (stippled line) crosses one.

Performing functional principal component analysis (FPCA), the three first FPC curves explained 90.7, 6.0 and 2.2% of the total variation between the individual curves, respectively; in sum almost 99% of the total observed variation. These three FPC curves are shown in Figure 3.

The first and by far most dominant FPC curve (FPC1) mainly represents the general level of the individual temporal FM curves relative to the overall temporal mean for all women. A high positive score on FPC1 implies longer than average counting times and a large negative score implies shorter than average counting times. Included in FPC1 is also a small increase in counting times the very last days before birth.

The second FPC curve (FPC2) relates to a linear increase or decrease in counting times as the pregnancy proceeds; women with a high score on FPC2 will have a tendency towards increasing counting times as the pregnancy proceeds, while women with a large negative score will have a tendency towards decreasing counting times. A small plateau appears in the FPC2 curve the last days prior to birth.

The third FPC curve (FPC3) has an inverted U-shape, and high scores on FPC3 indicate higher than average counting times in the mid of the counting period, while high negative scores implies shorter counting times in the mid period, compared to what is to be expected for that given general level.

**Figure 4:**
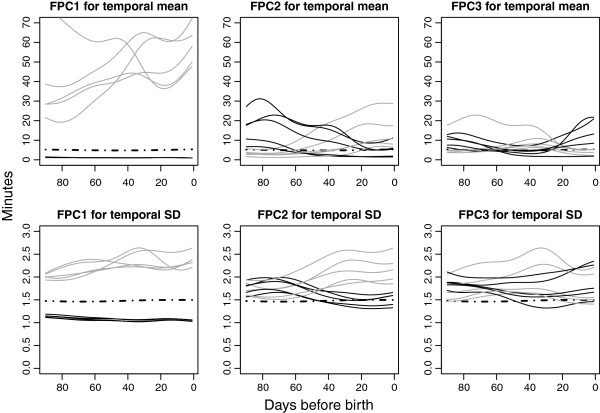
**Individual curves for women with largest positive and negative functional principal component scores.** Individual curves for the women with the five largest positive (black) and five largest negative (grey) scores for each of the three FPCs for smoothed fit and for residuals. Overall mean superimposed (dotted line).

Smooth temporal FM curves for the women with the five highest and five lowest scores for each of the three FPCs are shown in Figure 4. These individual FM count curves highlight the interpretations “General FM count level”, “Linear trend” and “U-shape”. FPCA extracts the fundamental temporal features from which the individual charts can be reconstructed. These main FPCAs are simple functions or “building blocks” with meaningful clinical interpretations. Individual charts would naturally be represented by some, or all, of the FPCAs to varying extent; a chart with a high mean, i.e. a high score on FPC1, could also have a large increase in counting times towards the end of the pregnancy, i.e. a high score on FPC2, as well as considerable scores on FPC3 through FPC10. Thus, it is consequently expected that the observed FM charts have more detail. Although miniscule for most women, the increased counting time just prior to birth in FPC1 becomes prominent in women with very large positive scores on this FPC, i.e. women with overall long counting times. The same applies to the small plateau in the FPC2 curve in the last days prior to birth.

The FPCs representing women's deviations from their own smooth means can be interpreted in a similar fashion. The three first FPC curves explain 86.6, 7.0 and 4.3% of the total variation in the residuals, respectively; in sum almost 98% of the observed variation. These FPCs are shown in Figure 3.

The first residual FPC curve is by far the most dominant, and represents the overall level for the temporal residuals. The second residual FPC curve relates to increasing or decreasing residual variability in FM counting times approaching birth, while the third residual FPC curve is an inverted U-shape, representing more variability in the beginning and end of the counting period compared to the middle.

Fitted smooth temporal FM curves for women with the five highest and five lowest scores for each of the three FPCs for temporal residuals are shown in Figure 4. Again, the interpretations “General FM count variability level”, “Linear trend” and “U-shape” are highlighted.

### Results from multiple regression analyses

**Figure 5:**
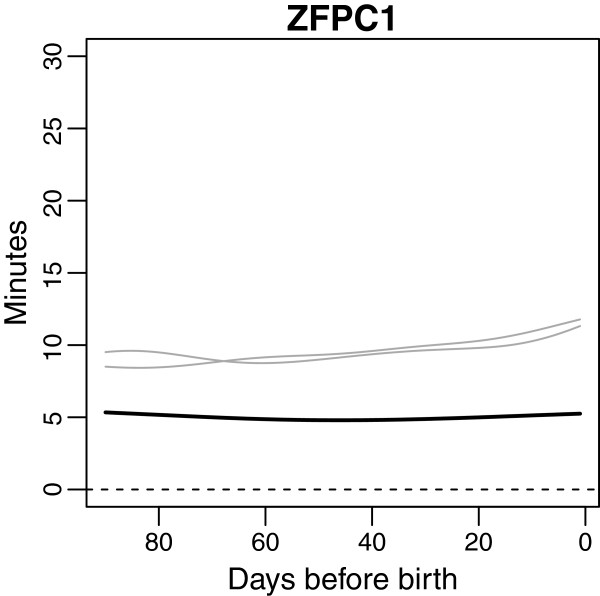
**Smooth curve fits corresponding to one standard deviation above the overall temporal mean.** Smooth curve fits for two women (grey solid line) with functional principal components scores (FPC1) corresponding to one standard deviation above the overall temporal mean for all women (black solid line).

The multiple linear regression results for the association between the variation in the three main FPC for the smoothed temporal mean and various pregnancy characteristics are presented in Table 2. Maternal obesity was significantly positively associated with scores on FPC1, and negatively associated with scores on FPC2, i.e. longer counting times with a decreasing trend as pregnancy advances. However, the effect of obesity on FPC1 was small, only 0.25, corresponding to one fourth of a standard deviation. For illustration, Figure 5 shows smooth FM curves for two women with FPC1 scores corresponding to one standard deviation above the overall temporal mean for all women. One standard deviation corresponds to approximately five minutes higher counting times. The effect of obesity is therefore miniscule, reflecting less than two minutes. Maternal overweight was negatively associated with FPC3, i.e. shorter counting times in the mid period. Anterior placental site was negatively associated with scores on both FPC2 and FPC3, meaning shorter counting times towards birth. Gestational age was significantly positively associated with both FPC1 and FPC3, implying higher counting times with an increase in the mid period.

**Table 2:** Linear regression with functional principal component scores for the mean as dependent variable

	**FPC1 for mean**				**FPC2 for mean**				**FPC3 for mean**			
	**Univariate linear regression**		**Multiple linear regression**		**Univariate linear regression**		**Multiple linear regression**		**Univariate linear regression**		**Multiple linear regression**	
	**Effect (95% CI)**	**p-value**	**Effect (95% CI)**	**p-value**	**Effect (95% CI)**	**p-value**	**Effect (95% CI)**	**p-value**	**Effect (95% CI)**	**p-value**	**Effect (95% CI)**	**p-value**
Maternal BMI ^I^ categorized												
Overweight ^II^	0.04	0.173	0.11	0.162	−0.18	0.097*	−0.13	0.093	−0.11	0.029**	−0.17	0.028**
	(−0.28,1.57)		(−0.04,0.26)		(−0.39,0.03)		(−0.28,0.02)		(−0.21,-0.01)		(−0.31,-0.02)	
Obesity ^III^	0.09	0.007**	0.25	0.006**	−0.06	0.046**	−0.22	0.014**	0.02	0.711	0.02	0.824
	(0.04,2.59)		(0.07,0.42)		(−0.51,-0.01)		(−0.40,-0.04)		(−0.09,0.14)		(−0.15,0.20)	
Primiparity	−0.03	0.353	−0.05	0.383	−0.13	0.119	−0.11	0.082	0.02	0.459	0.06	0.355
	(−1.09,0.39)		(−0.18,0.07)		(−0.30,0.03)		(−0.23,0.01)		(−0.05,0.11)		(−0.06,0.18)	
Anterior placental site^b^	0.04	0.183	0.10	0.127	−0.15	<0.001***	−0.32	<0.001***	−0.07	0.026**	−0.15	0.016**
	(−0.24,1.25)		(−0.03,0.22)		(−0.60,-0.27)		(−0.44, -0.19)		(−0.17,-0.11)		(−0.27,-0.03)	
Gestational age, days	- 0.04	0.012**	−0.01	0.006**	0.05	0.111	0.06	0.098	0.06	0.038**	0.08	0.021**
	(−0.08,-0.01)		(−0.17,-0.03)		(−0.00,0.11)		(−0.01, 0.13)		(0.00,0.01)		(0.00,0.15)	

Level of statistical significance * < 0.1, **< 0.05, ***< 0.001.

^I^ Body Mass Index, kg/m^2^.

^II^ Maternal overweight (25≤BMI<30) versus maternal normal or underweight (BMI <25.00).

^III^ Maternal obesity (30≤BMI) versus maternal normal or underweight (BMI <25.00).

^b^ Predominantly anterior placental site reported from routine ultrasound examination in pregnancy week 18.

Linear regression with scores on the functional principal components for the smooth curve fits for the mean as the dependent variable and basic pregnancy characteristics as explanatory variables.

Pregnancy characteristics were not significantly associated with the FPCs for residuals, i.e. each woman’s deviations from her own temporal mean (Additional file 5: Table S2). We found no significant association between long counting times (FPC1 for temporal mean) and large residuals (FPC1 for temporal residuals).

## Discussion

It is well acknowledged that quantitative limits for DFM perform poorly for screening purposes, indicating the need for further refinement [11,28]. Our study, as far as we know, is the first to extract individual temporal patterns from FM chart data. For this purpose, we have used functional data analysis (FDA) and functional principal components analysis (FPCA). Recognizing that extreme observations were removed before FDA, we found that almost all of the observed variation between women’s smoothed temporal FM curves was accounted for by mere three temporal components; a general FM count level, a linear trend, and a U-shape. These components can be readily interpreted in a biological context.

Fetal activity must be seen as a longitudinal process, as its temporal pattern provides important information. However, previous FM counting studies have mainly focused on fixed limits for DFM and their ability to identify risk [5]. Analyses of patterns in FM counting charts have mostly been restricted to healthy pregnancies aiming to define limits of normality [21,22,33]. These studies have, with few exceptions [20,34], focused on group averages and deviations from these [14,21,22], ignoring that observations from the same woman are naturally ordered in time, and strongly correlated. The conclusions from these studies may therefore be of limited value as key characteristics of FM chart data is unaccounted for. Direct comparisons of our outcomes with previous research may therefore be misleading.

A central element of FDA is fitting a smooth curve to the actual observations, effectively separating the underlying signal from the uninformative “noise”, e.g. natural day-to-day variation not reflecting any physiological change. As the natural, and random, variation in the counting process is often relatively high, a strong smoothing effect, as we see in our analysis, was expected.

Somewhat surprisingly, we did not find a statistically significant association between higher overall mean FM count and high SD, i.e. a woman’s smooth temporal mean and her day-to-day deviations from this temporal mean. For women with a strong increasing, linear trend, such as woman 7 in Figure 2, the crude, overall point-mean will be a poor representation of her temporal pattern, and the accompanying SD will be unrealistically high. However, when considering her temporal mean, the accompanying temporal SD is actually very low. Previous point-wise results will therefore be biased or outright misleading. Indeed, a crude, overall mean of 21 minutes does not capture the linear trend, and the corresponding SD of 9 minutes is a gross overestimate. Moreover, FM charts with comparable mean counting times may hide fundamentally different temporal patterns. The crude, overall mean (SD) for woman 3 in Figure 2 is 24 (16) minutes, similar to woman 7.

Although previous studies have rightly recognized the potential limitations of point-wise measures [20,33,35], none have provided meaningful alternatives. Our statistical approach demonstrates how temporal patterns in FM charts hold valuable information for the interpretation of relevant counting measures, and how this can be overlooked when not taking the temporal nature of FM chart data into account. The results indicate that conclusions from previous studies ought to be revisited.

Our results are consistent with previous research in two central areas. First, there is considerable variation in FM *between* pregnancies, but lower variation *within* pregnancies [19,20,36]. Second, pregnancy characteristics may explain some of the variation in perceived FM between pregnancies [19,28,36-38].

By far, the differences in the general level of the fitted temporal FM curves accounted for most of the variation between women. This may simply reflect that activity level between fetuses varies. However, it has also been suggested that women may differ in their ability to perceive FM [39].

Previous studies on the effect of maternal characteristics on women’s ability to perceive FM have not reached clear conclusions. One typical approach has been to compare ultrasound observed FM with those perceived by the mother and explore how these vary with maternal characteristics [39]. However, most of these studies did not account for the high correlation of observations within pregnancies. They were also small, with divergent results [39].

Another approach has been to compare maternal characteristics of women presenting spontaneously with DFM with reference groups [38,40], whereas FM counting studies have, with few exceptions [19,28], mainly reported whether maternal characteristics have been associated with various fixed alarms [19,36]. They have not explored the association between FM counting patterns and maternal characteristics. Thus there are few studies available to compare with our results.

Overweight and obese women more often report DFM [38]. They are also at increased risk of severe pregnancy complications [41]. However, since many have favorable outcomes [38,40], it has been suggested that the perceived DFM reflects reduced sensitivity to FM from excess adipose tissue rather than fetal compromise. There is to date no firm knowledge to disentangle these effects [39].

In line with previous studies [19,28], we found that maternal obesity was significantly associated with higher counting times compared to the reference group (BMI<25). Yet the effect was very small. Note that the effect of maternal BMI was related to obesity and not overweight. Thus, our study suggests that FM counting is applicable also for overweight and obese women. This is important since these women represent a large and growing risk group for obstetric complications in high income countries [41]. Our result is contrasting a previous study stating that DFM may have greater diagnostic significance in normally weighing women [40]. This former study is influential as it is cited in a recent Cochrane review on management strategies for women perceiving DFM [42].

Anterior placental site has been reported to decrease a woman’s perception of FM prior to 28 weeks of gestation [37]. We found anterior placental site to be significantly associated with a moderate down-towards-birth pattern (FPC2) and with a U-shaped pattern (FPC3), combining the gradual decrease in counting time with a small increase in late gestation. However, for most women, this effect was small similar to what was found for the general level. As seen in Figure 4, even for the women with the five largest positive and the five largest negative scores, the individual curves show modest changes in terms of minutes. Parity is reported not to influence FM counting once quickening is reached [19,23,28]. This corresponds with our findings.

We aligned our data from birth and 90 days backwards, so that we could capture FM counting patterns approaching delivery. Contrary to previous studies reporting that counting times remain constant [19,28,33] or increase [20-22] with advancing gestation, we found increasing gestational age to be associated with shorter counting times (FPC1). However, as mentioned, direct comparisons with previous studies may be misleading, as these tend to not account for the intra-woman correlation in FM chart data. Importantly, with this counting method, it is not normal for women to perceive DFM in late gestation. Note that the statistically significant associations in this study reflect overall relatively small effects.

We included pregnancies from a total population in our analyses. FPCA sequentially extracts the various temporal patterns where the variation between women is the largest, second largest and so on. As unfavorable birth outcomes are relatively rare, (possible) temporal patterns related to such pregnancies would not be common in a large group of women, consequently ranging low in relative importance of the FPCA. Extracting a large amount of FPCAs would capture these patterns, but these will, by mathematical construction of the PCA, not affect the main results, i.e. the main temporal patterns.

Three limitations need to be mentioned. Firstly, the compliance with daily counting was towards the lower end of the 55-97% range previously reported [12,14,15,21,28,34]. Our recruitment rate was higher than in previous reports [15,28], which might have caused a higher drop-out rate. Moreover, mothers were asked to count FM from pregnancy week 24, earlier than in previous studies [12,14,15,28,34], which might have caused reporting fatigue. Secondly, our sample appears to be skewed towards healthier pregnancies, similar to what have been reported previously [15,28]. FM counting may be more appealing to mothers with active babies, since they are then reassured about the baby’s well-being within a short time. Therefore, both counting times and day-to-day variability may be underestimated compared to a total population. Thirdly, the FDA approach was well-suited for extracting individual temporal FM counting patterns and for exploring their associations with pregnancy characteristics. However, it was not suitable for capturing the rapid temporal changes introduced by the spikes, i.e. sudden long counting times relative to the body of the woman's observations. Such "alarms" may reflect acute changes to fetal well-being, and merits further investigation. However, a different statistical approach is required for spikes to be captured in long time series. Spikes occur seemingly randomly throughout pregnancy as illustrated in Figure 1 and occur in both healthy and riskier pregnancies [28]. These spikes would tend to be averaged out with our FDA approach. Time has escaped the “fixed” limits for DFM. Before studying pathological FM counting patterns, future analyses should explore extreme observations in the FM chart, as well as other time-dependent out-of-the-ordinary observations, when modeling FM count data.

There seemed to be reporting fatigue in the FM charts, with compliance rates falling towards term. Previous FM counting studies have consistently reported that continued encouragement from health care providers yields the most complete findings [34,43]. Before we can expect higher acceptance rates, FM counting must prove useful to both women and care providers. Hence, better information about normal FM and how to interpret FM counting patterns is needed. Although not applicable on an individual level, we have with this comprehensive statistical approach taken an important first step in identifying temporal patterns in FM charts.

Our results carry important clinical messages. A perceived change in FM should not be attributed to a woman’s maternal characteristics or placental location, but rather be interpreted as a true change in FM, potentially indicating fetal compromise. This should be clarified in published guidelines [44]. Further, maternal characteristics or anterior placental site do not seem to be incompatible with FM counting. Finally, the wide-spread notion that fetal activity decreases in late pregnancy is refuted. With this counting method, a decrease in FM in late pregnancy is not normal. This is a core component of information that should be provided to pregnant women [45].

## Conclusions

We have successfully extracted the main temporal patterns in FM counting data, both overall and for individual women. Results from previous studies, which do not take intra-woman correlation of counting observations into consideration, might need to be revisited. Overall, pregnancy characteristics explained little of the variation in temporal FM counting patterns, implying that perceived DFM should be interpreted independent from these characteristics.

## Appendix A, Statistical procedures

Outlying observations were identified by removing the estimated underlying time series trend in each FM chart, and assessing interquartile range (IQR) for the remaining residuals. IQR above 1.5 was used as the cutoff for being an outlier [46].

We simultaneously performed missing imputation, fitting of smooth curves to each woman’s FM chart, and estimation of general temporal patterns across women by calculation of functional principal components, using a Bayesian approach [29,47].

To completely specify the Bayesian model, one needs to provide prior distributions for the model parameters. We used independent Gamma (10^-3^, 10^-3^) priors for the variances, and ten eigenfunctions. We ran 1500 simulations, and disregarded the first 500 as burn-in. This ensured an R^ of approximately 1 for all parameters, indicating convergence [48].

Credibility intervals (CrI) are the Bayesian parallel to confidence intervals (CI) to assess estimation uncertainty. The methodology applied returns 95% CrIs for the fitted functional objects to the individual FM count data [29].

The Bayesian version of functional principal component analysis applied in this work is described in detail in Crainiceanu and Goldsmith [29].

They also give the general WinBugs code needed to run the analysis. We recommend for the interested reader to obtain more detailed information on the applied FPCA methodology in the sited reference. The statistical analyses were done in R 2.12 [49].

For the FDA we used function R2Winbugs [50] to perform the Bayesian simulation in WinBugs [51,52].

## Competing interests

This project is funded in whole by the Research Council of Norway. The authors declare they have no competing interests.

## Authors’ contributions

All three authors have contributed to this scientific work and have approved the final version of the manuscript. BAW has been responsible for data collection and for scientific interpretation of the FDA/PCA results, for regression analyses and for writing and revising the manuscript. JR has been responsible for the FDA/FPCA analyses, presentation of the various plots, and for writing the statistics section and the Appendix, and revising the manuscript. JFF had the original idea for the study, has contributed to scientific interpretation of the FDA/FPCA and for revising the manuscript.

## Pre-publication history

The pre-publication history for this paper can be accessed here:

http://www.biomedcentral.com/1471-2393/12/124/prepub

## Supplementary Material

Additional file 1 **Figure S1.** Flowchart of recruitment. Click here for file

Additional file 2 **Appendix 1.** Counting protocol. Click here for file

Additional file 3 **Appendix 2.** Fetal movement chart. Click here for file

Additional file 4 **Table S1.** Characteristics of pregnancies excluded from analyses and those included. Click here for file

Additional file 5 **Table S2.** Linear regression with scores on the functional principal components (FPC) for smooth curve fits for residuals (SD) as the dependent variable. Click here for file
