# Supplementary figures and images for: Temporal patterns in count-to-ten fetal movement charts and their associations with pregnancy characteristics: a prospective cohort study
Source: BMC Pregnancy Childbirth. 2012 Nov 6;12:124. doi: 10.1186/1471-2393-12-124 (PMC3542088; doi:10.1186/1471-2393-12-124)

Flow chart for recruitment

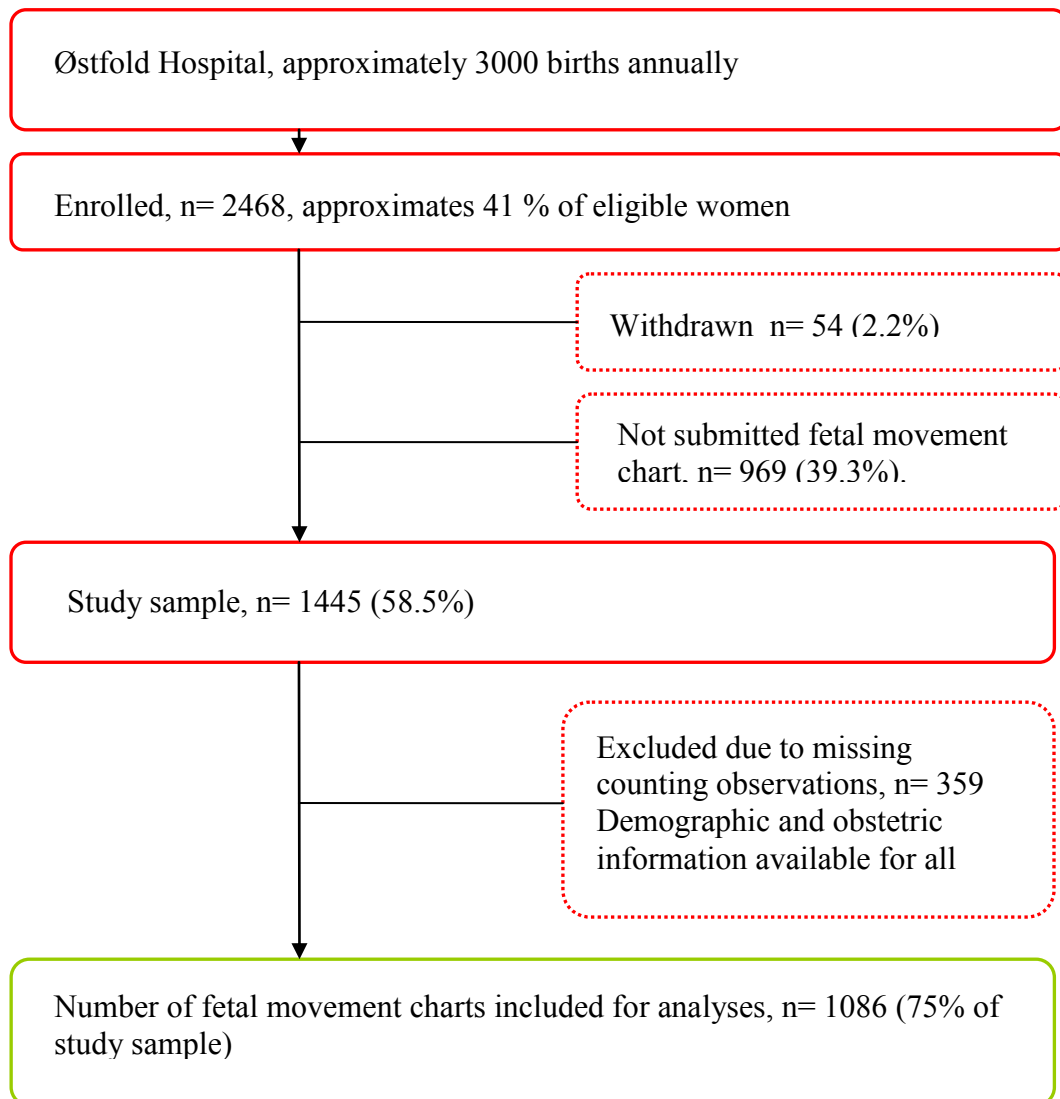

Supplement: Additional file 1 — Figure S1. Flowchart of recruitment. [file 1471-2393-12-124-S1.pdf]
