# Supplementary material for: Temporal patterns in count-to-ten fetal movement charts and their associations with pregnancy characteristics: a prospective cohort study
Source: BMC Pregnancy Childbirth. 2012 Nov 6;12:124. doi: 10.1186/1471-2393-12-124 (PMC3542088; doi:10.1186/1471-2393-12-124)
Supplement: Additional file 2 — Appendix 1. Counting protocol. [file 1471-2393-12-124-S2.pdf]

Count with me

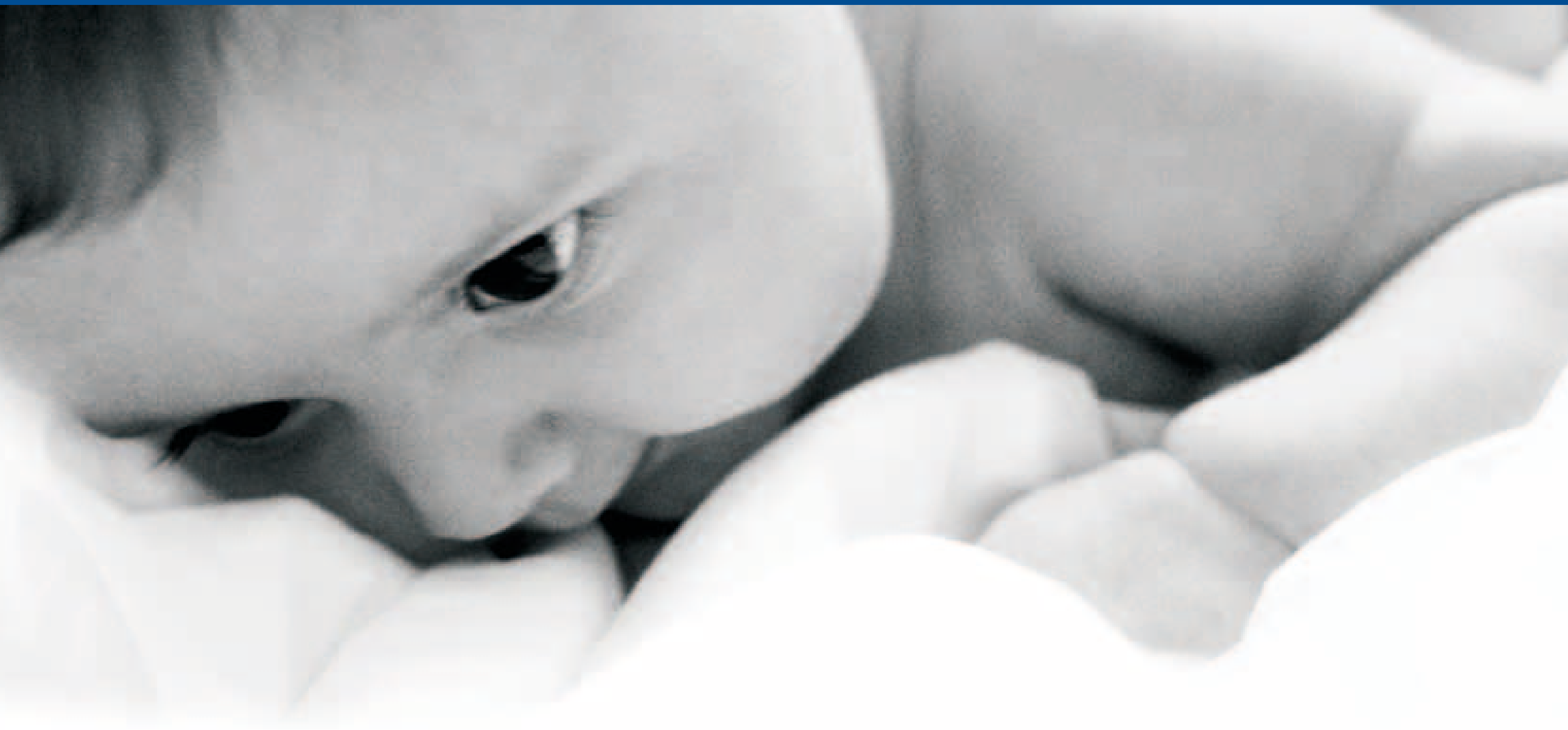

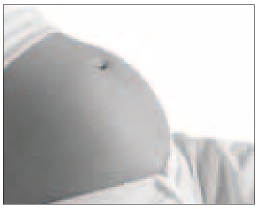

CAN KICK COUNTING INCREASE OUR KNOWLEDGE ABOUT THE BABY'S HEALTH DURING PREGNANCY?

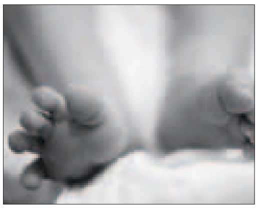

The research project *Count with me*
is a collaboration between
the Norwegian Institute of Public Health and Østfold Hospital Trust

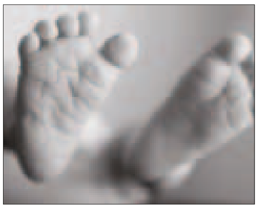

www.fhi.no/tellmedmeg
www.fhi.no/countwithme

Norwegian Institute of Public Health

SYKEHUSET ØSTFOLD

Count with me

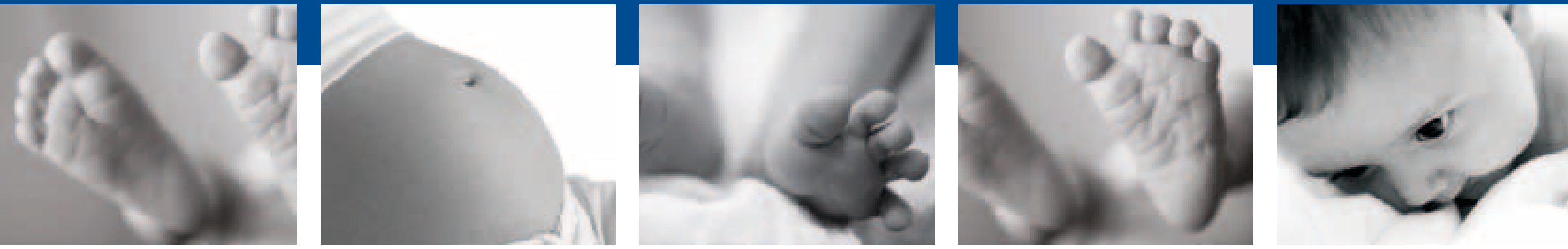

You can find this
information in English:
www.fhi.no/countwithme

Thank you for choosing to participate in the *Count with me* project. *Count with me* is a collaborative project between The Norwegian Institute of Public Health and Østfold Hospital Trust.

This folder contains information about fetal movements, what they mean and what participation in *Count with me* implies. You will also find instructions on how to complete the kick count form from pregnancy week 24 and up to birth.

This folder contains:

- Information about the *Count with me* project
- Information about fetal activity
- Information about decreased fetal activity
- Information on how to complete the kick count form
- Project contact information
- Kick count form
- Information for your midwife/doctor
- Referral form for placental examination (to be brought to the maternity unit)
- Postage-paid envelope for return of the kick count form after delivery

Norwegian Institute of Public Health

SYKEHUSET
ØSTFOLD

Information about the *Count with me* project

Count with me is part of an international research collaboration on fetal activity, Femina (Fetal Movement Intervention Assessment). The *Count with me* project aims to increase knowledge on fetal activity and what it tells us about the baby's health. Generally we have limited knowledge of what constitutes normal fetal activity, particularly prior to pregnancy week 28. With *Count with me* we wish to provide better information to pregnant women on what is perceived as normal fetal activity and which changes in the movement patterns are associated with risk. The project aims to develop individualized kick count forms that can help pregnant women assess their baby's activity pattern.

Regular pregnancy check-ups will help you monitor your baby's well-being, regardless of whether you participate in the study or not.

WHAT DO YOU HAVE TO DO?

- Count kicks once a day from day 1 in pregnancy week 24. Kicks include all kinds of movements.

- Hand over the information prepared for your doctor/midwife, to inform them of your participation in the study.

- You will find information in the folder about what to do if you experience significant changes in your baby's movement patterns. If you contact antenatal care services because you are worried that the baby is kicking less than normal, we ask you to answer the small questionnaire on the back on the kick count form. Should you contact antenatal care services on several occasions, please complete the questionnaire each time. If you are examined because your baby is less active than normal we ask you to place the red sticker marked *Tell med meg* (attached) on the form "Histologi". This is a referral form for placental examination.

- Keep the referral form with your pregnancy record and bring it to the maternity unit when you are in labour, so they know you are participating in the project.

- After delivery, please return the completed kick count form, either to a midwife or a mailbox marked "*Tell med meg*" at the hospital. You can also send it to the Norwegian Institute of Public Health in the enclosed reply envelope.

Information about fetal activity

GET TO KNOW YOUR BABY

After birth you will spend a lot of time taking care of your baby. During pregnancy, routine check-ups will help you to monitor the baby's well-being, but you as the mother are in the best position to get to know the baby before birth. We encourage you to devote a little time each day during pregnancy to get to know your baby by its movements.

There are great variations among healthy babies as to how often and how hard they kick. Kicking includes all types of movements. Some mothers feel kicks less obviously than others. If the placenta is at the front of the uterus or you are overweight, kicks are not felt as easily. Many pregnant women feel movements best when they are lying down and least when they are standing, walking or are busy with something. The majority of babies are most active in the evening or early morning.

HOW DOES THE BABY MOVE?

The baby is active throughout the pregnancy and makes large and small movements:

- Breathing movements, to expand the lungs. Your baby is practicing for life outside the womb. You will not notice these small movements.
- Small gripping movements, thumb-sucking or bending or stretching of fingers and toes. You will rarely notice these movements.
- The baby has hiccups. You might recognise these as regular small jerks.
- Larger body movements. These may occur when you change position from standing to lying. The baby can react to your movements and shift position. Most of the baby's kicks and pushes are easily noticeable.

The baby sleeps frequently during the day, and will normally not move during sleep. Towards the end of the pregnancy, the baby's sleep periods get longer, but even near due date it is rare that the baby sleeps more than an hour at a time. The way you recognise movements will change during pregnancy, but when the baby is awake, you should feel just as much activity in late pregnancy.

HOW MUCH SHOULD THE BABY KICK?

There is no clear definition of what is considered normal fetal activity and there are great variations between pregnancies. Most importantly, you should not experience considerable and persistent reductions in activity compared to normal levels for your baby. A kick count form will allow you to get familiar with the normal movement pattern for your baby.

The placenta transfers everything the baby needs from you to thrive. As long as supplies from the placenta are good, the baby will kick regularly. Sometimes the blood flow in the placenta can be affected due to different causes, e.g. smoking. If the placenta is not functioning well, the baby has to save energy, thus growth can be limited and the kicks will diminish.

Information about decreased fetal activity

WHAT IS DECREASED FETAL ACTIVITY AND WHAT SHOULD YOU DO?

After using the kick count form for a week or two, you will probably see that the kicking varies a little from day to day. For the most part, however, the days will appear to be similar. This way you will know what is normal activity for your baby, and this will continue for a thriving baby.

If you are worried about your baby, regardless of reason, you should seek advice and help from your doctor or midwife. If you are concerned because your baby is less active as the weeks go by, you should bring the kick count form to your next pregnancy check-up.

In some cases you should contact the maternity unit directly:

- If your baby does not kick one day, you should never wait until the next day.
- If your baby kicks progressively less during the day/days and you feel decreased activity.

If you are in doubt about what is low activity, you should know it is rare that a healthy baby kicks less than 10 kicks within a two-hour period when you know the baby is usually active. If you feel the baby has kicked scarcely the whole day, you should contact the maternity unit. If you are unsure of your baby's activity on a given day, before you started that day's counting, you should be alert. Make sure you count again within the next 12 hours, and contact the maternity unit if the results are repeated.

HAVE YOU CONTACTED HEALTH CARE PERSONNEL BECAUSE YOU HAVE BEEN WORRIED ABOUT DECREASED FETAL ACTIVITY?

- If so, we ask you to complete the small questionnaire on the back of the kick count form. Should you contact health care personnel on several occasions, please complete the questionnaire each time.

HAVE YOU BEEN EXAMINED BECAUSE YOU HAVE BEEN WORRIED ABOUT DECREASED FETAL ACTIVITY?

- If so, we ask you to complete the small questionnaire on the back of the kick count form. Should you contact health care personnel on several occasions, please complete the questionnaire each time.
- Bring the form to the maternity unit when you are in labour.

How to complete the kick count form?

- Fill in your due date as determined by ultrasound.
- Start counting kicks when you have reached day 1 in pregnancy week 24.
- Fill in the date you start counting.
- Count kicks/movements every day.
- Start counting, preferably within the same two-hour period every day. Choose a time of day when it suits you to set aside a little time and when you know the baby is usually active. If the usual time is not convenient on one day, choose another time on the same day.
- Fill in the time period you are usually counting.
- Count to 10. Record the time from you feel the first kick/movement (so you know the baby is awake) and until you have counted an additional nine movements. All movements count as kicks, but many simultaneous movements count as one kick. Hiccups should not be counted as movements.
- It usually takes less time if you lie/sit down, relax and pay attention. If the baby is asleep, you can try to wake it by pressing your stomach lightly or by drinking something cold.
- Record the number of minutes in the appropriate 5-minutes box on the kick count form.

If you follow this procedure, it takes approximately 10 minutes to count 10 kicks/movements.

Use a blue or black pen. Record the number of minutes it took to count 10 kicks/movements.

How to complete the kick count form

Use a blue or black pen.

For your convenience, fill in weekday and date in the appro-priate box.

After counting 10 kicks/movements, record the number of minutes in the appropriate 5-minutes box on the kick count form.

Examples:

If it took 7 minutes, write 7 in the 6-10-minutes box.

If it took 1 hour and 5 minutes, write 65 in the 60-65 minutes box.

Week 24

Weekday:	We	Th	Fr	Sa	Su	Mo	Tu
Date:	7/3	8/3					
Minutes:							
0-5							
6-10	7						
11-15							
16-20							
21-25							
26-30							
31-35							
36-40							
41-45							
46-50							
51-55							
56-60							
61-65		65					
66-70							
71-75							
76-80							
81-85							
86-90							
91-95							
96-100							
101-105							
106-110							
111-115							
116-120							
More than 2h? Record exact no of min.							

Send in the kick count form after birth

After delivery, please return the completed kick count form, either to a midwife or a mailbox marked *Tell med meg* at the hospital. You can also send it to the Norwegian Institute of Public Health in the enclosed reply envelope. It is important that you return your form, regardless of whether you have completed it partially or fully.

If you send the kick count form by mail, please use the postage paid reply envelope or send it to:

The Norwegian Institute of Public Health
Department of Genes and Environment
Count with me
P.O. Box 4404 Nydalen
0403 Oslo

Contacts

Please contact us if you are unsure about how to count, or if you have other queries.

**Østfold Hospital Trust**	**Norwegian Institute of Public Health**	
Tone Larsen<br>Project Midwife<br><br>e-post: tonlar@so-hf.no<br>Telephone: 69 86 13 05<br>Hours: 09 -15	Jorid Eide<br>Project Coordinator, Midwife<br><br>e-post: tellmedmeg@fhi.no<br>Telephone: 902 74 958<br>Hours: 09 -15	Brita Askeland Winje<br>Project Manager, PhD student<br><br>e-post: tellmedmeg@fhi.no<br>Telephone: 902 74 958<br>Hours: 09 -15

Project Administrator: Frederik Frøen, MD, Norwegian Institute of Public Health.

Find more information on our webpage: www.fhi.no/countwithme

Photo: Pregnant, Istock • Child: PhotoAlto/Luth • Feet: www.colourbox.com

Norwegian Institute of Public Health

SYKEHUSET ØSTFOLD
