# Supplementary material for: Temporal patterns in count-to-ten fetal movement charts and their associations with pregnancy characteristics: a prospective cohort study
Source: BMC Pregnancy Childbirth. 2012 Nov 6;12:124. doi: 10.1186/1471-2393-12-124 (PMC3542088; doi:10.1186/1471-2393-12-124)
Supplement: Additional file 3 — Appendix 2. Fetal movement chart. [file 1471-2393-12-124-S3.pdf]

Kick count form, page 1

I started to count today (dd.mm.yy) __ __ . __ __ . __ __   Due date by ultrasound (dd.mm.yy) __ __ . __ __ . __ __   I usually count between (hours) __ __ . __ __   and (hours) __ __ . __ __

How to complete the kick count form

Use a blue or black pen.

For your convenience, fill in weekday and date in the appropriate box.

After counting 10 kicks/movements, record the number of minutes in the appropriate 5-minutes box on the kick count form.

Examples:

If it took 7 minutes, write 7 in the 6-10-minutes box.

If it took 1 hour and 5 minutes, write 65 in the 60-65 minutes box.

Week 24

	We	Th	Fr	Sa	Su	Mo	Tu
Date:	7/3	8/3					
Minutes:							
0-5							
6-10	7						
11-15							
16-20							
21-25							
26-30							
31-35							
36-40							
41-45							
46-50							
51-55							
56-60							
61-65		65					
66-70							
71-75							
76-80							
81-85							
86-90							
91-95							
96-100							
101-105							
106-110							
111-115							
116-120							
More than 2h? Record exact no of min.							

Fill in your study number if you are participating in *Count with me.*

	Week 24	Week 25	Week 26	Week 27	Week 28
Weekday:					
Date:					
Minutes:					
0-5					
6-10					
11-15					
16-20					
21-25					
26-30					
31-35					
36-40					
41-45					
46-50					
51-55					
56-60					
61-65					
66-70					
71-75					
76-80					
81-85					
86-90					
91-95					
96-100					
101-105					
106-110					
111-115					
116-120					
More than 2h? Record exact no of min.					

Continue registration on page 2

Kick count form, page 2

	Week 29	Week 30	Week 31	Week 32	Week 33	Week 34	Week 35
Weekday:							
Date:							
Minutes:							
0-5							
6-10							
11-15							
16-20							
21-25							
26-30							
31-35							
36-40							
41-45							
46-50							
51-55							
56-60							
61-65							
66-70							
71-75							
76-80							
81-85							
86-90							
91-95							
96-100							
101-105							
106-110							
111-115							
116-120							
More than 2h? Record exact no of min.							

Continue registration on page 3

Kick count form, page 3

	Week 36							Week 37							Week 38							Week 39							Week 40							Week 41							Week 42						
Weekday:																																																	
Date:																																																	
Minutes:																																																	
0-5																																																	
6-10																																																	
11-15																																																	
16-20																																																	
21-25																																																	
26-30																																																	
31-35																																																	
36-40																																																	
41-45																																																	
46-50																																																	
51-55																																																	
56-60																																																	
61-65																																																	
66-70																																																	
71-75																																																	
76-80																																																	
81-85																																																	
86-90																																																	
91-95																																																	
96-100																																																	
101-105																																																	
106-110																																																	
111-115																																																	
116-120																																																	
More than 2h? Record exact no of min.																																																	

REMEMBER TO RETURN YOUR KICK COUNT FORM AFTER DELIVERY. THANK YOU FOR YOUR PARTICIPATION.

HAVE YOU CONTACTED HEALTH CARE PERSONNEL DUE TO DECREASED FETAL ACTIVITY?

If so, please complete the questions below. Should you contact health care personnel on several occasions, please complete the questionnaire each time (use an extra sheet of paper if necessary).

1. Have you CONTACTED health care personnel because you have been worried about decreased activity? If so, when: ☐☐.☐☐.☐☐ (dd mm yy)

2. What did you DO?

I waited for ☐☐ hours before I CALLED:

- ☐ Antenatal care services/health clinic
- ☐ Maternity unit
- ☐ Other____________________

I waited for ☐☐ hours before I VISITED

- ☐ Antenatal care services/health clinic
- ☐ Maternity unit
- ☐ Other____________________

3. If you were EXAMINED, how did you perceive the baby's activity level at that time?

- ☐ The activity level was normal
- ☐ The activity level was still decrased

1. Have you CONTACTED health care personnel because you have been worried about decreased activity? If so, when: ☐☐.☐☐.☐☐ (dd mm yy)

2. What did you DO?

I waited for ☐☐ hours before I CALLED:

- ☐ Antenatal care services/health clinic
- ☐ Maternity unit
- ☐ Other____________________

I waited for ☐☐ hours before I VISITED

- ☐ Antenatal care services/health clinic
- ☐ Maternity unit
- ☐ Other____________________

3. If you were EXAMINED, how did you perceive the baby's activity level at that time?

- ☐ The activity level was normal
- ☐ The activity level was still decrased

1. Have you CONTACTED health care personnel because you have been worried about decreased activity? If so, when: ☐☐.☐☐.☐☐ (dd mm yy)

2. What did you DO?

I waited for ☐☐ hours before I CALLED:

- ☐ Antenatal care services/health clinic
- ☐ Maternity unit
- ☐ Other____________________

I waited for ☐☐ hours before I VISITED

- ☐ Antenatal care services/health clinic
- ☐ Maternity unit
- ☐ Other____________________

3. If you were EXAMINED, how did you perceive the baby's activity level at that time?

- ☐ The activity level was normal
- ☐ The activity level was still decrased

1. Have you CONTACTED health care personnel because you have been worried about decreased activity? If so, when: ☐☐.☐☐.☐☐ (dd mm yy)

2. What did you DO?

I waited for ☐☐ hours before I CALLED:

- ☐ Antenatal care services/health clinic
- ☐ Maternity unit
- ☐ Other____________________

I waited for ☐☐ hours before I VISITED

- ☐ Antenatal care services/health clinic
- ☐ Maternity unit
- ☐ Other____________________

3. If you were EXAMINED, how did you perceive the baby's activity level at that time?

- ☐ The activity level was normal
- ☐ The activity level was still decrased
