# Supplementary material for: Temporal patterns in count-to-ten fetal movement charts and their associations with pregnancy characteristics: a prospective cohort study
Source: BMC Pregnancy Childbirth. 2012 Nov 6;12:124. doi: 10.1186/1471-2393-12-124 (PMC3542088; doi:10.1186/1471-2393-12-124)
Supplement: Additional file 4 — Table S1. Characteristics of pregnancies excluded from analyses and those included. [file 1471-2393-12-124-S4.docx]

Additional Table 1, Characteristics of pregnancies excluded from analyses and those included.

|  |  | | **Excluded, due to low compliance** | **Included for analyses** |  |  |
| --- | --- | --- | --- | --- | --- | --- |
|  |  | | **n=359** | **n=1086** | **RR (95%CI)** | p^//^ |
|  |  | | n (%) | n (%) |  |  |
| **MATERNAL CHARACTERISTICS** | | |  |  |  |  |
|  |  | Maternal age ≥ 35 yrs | 41 (11.4) | 196 (18.0) | 0.6 (0.5-0.9) | 0.004 |
|  |  | Maternal obesity (BMI ≥ 30 kg/m^2^) | 51 (14.2) | 155 (15.0) | 1.0 (0.7-1.3) | 0.975 |
|  |  | Primiparity | 188 (52.4) | 574 (52.9) | 1.0 (0.9-1.1) | 0.873 |
|  |  | Daily/occasionally smoking 1.trimester | 42 (11.7) | 93 (8.5) | 1.4 (1.0-1.9) | 0.075 |
|  |  | Anterior placental site | 165 (46.0) | 479 (44.1) | 1.0 (0.9-1.2) | 0.536 |
| **CHARACTERISTICS OF THE NEWBORNS AND BIRTH OUTCOME** | | |  |  |  |  |
|  |  | Male gender | 190 (52.9) | 562 (51.7) | 1.0 (0.9-1.1) | 0.697 |
|  |  | Birth weight in grams (mean) | 3566 [595] | 3585 [524.5] | (mean diff: -19) | 0.567 |
|  |  | Low birthweight (<2500gr) | 13 (3.6) | 27 (2.5) | 1.5 (0.8-2.8) | 0.257 |
|  |  | Stillbirth (> 22 weeks, per 1000) | - | 3 [2.7/1000] |  |  |
|  |  | Preterm (22^0^-36^6^ weeks) | 21 (5.8) | 52 (4.8) | 1.2 (0.7-2.0) | 0.426 |
|  |  | Apgar score <7_5min_ | 6 (1.7) | 16 (1.5) | 1.1 (0.5-2.9) | 0.791 |

The table presents demographic and obstetric characteristics and birth outcome for women who were excluded from analyses due to low counting compliance (i.e. had more missing than reported observations, 3/7 = 43%), compared to those included
