# Supplementary material for: Temporal patterns in count-to-ten fetal movement charts and their associations with pregnancy characteristics: a prospective cohort study
Source: BMC Pregnancy Childbirth. 2012 Nov 6;12:124. doi: 10.1186/1471-2393-12-124 (PMC3542088; doi:10.1186/1471-2393-12-124)
Supplement: Additional file 5 — Table S2. Linear regression with scores on the functional principal components (FPC) for smooth curve fits for residuals (SD) as the dependent variable. [file 1471-2393-12-124-S5.docx]

Additional Table 2: Linear regression with scores on the functional principal components (FPC) for smooth curve fits for residuals (SD) as the dependent variable.

|  | FPC1 for SD | | | | FPC2 for SD | | | | FPC3 for SD | | | |
| --- | --- | --- | --- | --- | --- | --- | --- | --- | --- | --- | --- | --- |
|  | Univariate  linear regression | | Multiple  linear regression | | Univariate  linear regression | | Multiple  linear regression | | Univariate  linear regression | | Multiple  linear regression | |
|  | Effect  (95% CI) | p-value | Effect  (95% CI) | p-value | Effect  (95% CI) | p-value | Effect  (95% CI) | p-value | Effect  (95% CI) | p-value | Effect  (95% CI) | p-value |
| Maternal BMI ^I^, categorised |  |  |  |  |  |  |  |  |  |  |  |  |
| Overweight ^II^ | 0.03  (-0.09,0.25) | 0.369 | 0.05  (-0.10,0.20) | 0.535 | -0.01  (-0.04,0.03) | 0.882 | -0.02  (-0.17,0.13) | 0.791 | 0.02  (-0.10,0.20) | 0.698 | 0.04  (-0.11,0.19) | 0.582 |
| Obesity ^III^ | -0.00  (-0.20,0.19) | 0.961 | -0.03  (-0.20,0.15) | 0.762 | -0.01  (-0.04,0.04) | 0.832 | -0.03  (-0.21,0.15) | 0.751 | 0.05  (-0.04,0.31) | 0.124 | 0.13  (-0.05,0.30) | 0.158 |
| Primiparity | 0.01  (-0.12,0.15) | 0.813 | 0.02  (-0.10,0.14) | 0.752 | -0.05  (-0.05,0.01) | 0.115 | -0.11  (-0.23,0.02) | 0.092* | -0.03  (-0.19,0.05) | 0.272 | -0.07  (-0.20,0.05) | 0.240 |
| Anterior placental site *^b^* | 0.01  (-0.11,0.16) | 0.714 | -0.03  (-0.16,0.09) | 0.598 | 0.04  (-0.01,0.05) | 0.159 | 0.07  (-0.06,0.20) | 0.281 | -0.01  (-0.01,0.01) | 0.862 | -0.02  (-0.14,0.11) | 0.788 |
| Gestational age, days | -0.05  (-0.01,0.00) | 0.109 | -0.05  (-0.11,0.02) | 0.189 | 0.00  (-0.00,0.00) | 0.974 | -0.00  (-0.07,0.07) | 0.976 | 0.05  (-0.00,0.00) | 0.150 | 0.05  (-0.02,0.12) | 0.153 |
| FPC1 for mean^§^ | 0.04  (-0.01,0.02) | 0.233 | 0.05  (-0.01,0.11) | 0.123 | 0.01  (-0.00,0.00) | 0.881 | 0.00  (-0.06,-0.07) | 0.864 | 0.01  (-0.00,0.00) | 0.876 | -0.00  (-0.06,0.06) | 0.966 |
| FPC2 for mean | -0.06  (-0.09,0.00) | 0.062* | -0.05  (-0.11,0.01) | 0.089* | -0.07  (-0.02,-0.00) | 0.019** | -0.07  (-0.12,-0.00) | 0.039** | -0.05  (-0.01,0.00) | 0.133 | -0.04  (-0.10,0.02) | 0.207 |
| FPC3 for mean | -0.08  (-0.24,-0.04) | 0.007** | -0.08  (-0.13,-0.01) | 0.016** | 0.01  (-0.02,0.03) | 0.640 | 0.02  (-0.05,0.08) | 0.588 | -0.02  (-0.01,0.01) | 0.427 | -0.02  (-0.08,0.05) | 0.626 |

*Statistical significance * < 0.1, **< 0.05, ***< 0.001*

*^I^ Body Mass Index, kg/m^2^*

*^II^ Maternal overweight (25≤BMI<30) versus maternal normal or underweight (BMI <25.00)*

*^III^ Maternal obesity (30≤BMI) versus maternal normal or underweight (BMI <25.00)*

*^b^ Predominantly anterior placental site reported from routine ultrasound examination in pregnancy week 18*

*^§^ Functional principal component for the smooth curve fits for the mean*
